# Virological failure and all-cause mortality in HIV-positive adults with low-level viremia during antiretroviral treatment

**DOI:** 10.1371/journal.pone.0180761

**Published:** 2017-07-06

**Authors:** Olof Elvstam, Patrik Medstrand, Aylin Yilmaz, Per-Erik Isberg, Magnus Gisslén, Per Björkman

**Affiliations:** 1 Department of Translational Medicine, Clinical Infection Medicine Unit, Lund University, Malmö, Sweden; 2 Department of Translational Medicine, Clinical Virology, Lund University, Malmö, Sweden; 3 Institute of Biomedicine, Department of Infectious Diseases, Sahlgrenska Academy, University of Gothenburg, Gothenburg, Sweden; 4 Department of Statistics, Lund University, Lund, Sweden; University of Pittsburgh, UNITED STATES

## Abstract

**Objective:**

Although most HIV-infected individuals achieve undetectable viremia during antiretroviral therapy (ART), a subset have low-level viremia (LLV) of varying duration and magnitude. The impact of LLV on treatment outcomes is unclear. We investigated the association between LLV and virological failure and/or all-cause mortality among Swedish patients receiving ART.

**Methods:**

HIV-infected patients from two Swedish HIV centers were identified from the nationwide register InfCare HIV. Subjects aged ≥15 years with triple agent ART were included at 12 months after treatment initiation if ≥2 following viral load measurements were available. Patients with 2 consecutive HIV RNA values ≥1000 copies/mL at this time point were excluded. Participants were stratified into four categories depending on viremia profiles: permanently suppressed viremia (<50 copies/mL), LLV 50–199 copies/mL, LLV 200–999 copies/mL and viremia ≥1000 copies/mL. Association between all four viremia categories and all-cause death was calculated using survival analysis with viremia as a time-varying covariate, so that patients could change viremia category during follow-up. Association between the three lower categories and virological failure (≥2 consecutive measurements ≥1000 copies/mL) was calculated in a similar manner.

**Results:**

LLV 50–199 copies/mL was recorded in 70/1015 patients (6.9%) and LLV 200–999 copies/mL in 89 (8.8%) during 7812 person-years of follow-up (median 6.5 years). LLV 200–999 copies/mL was associated with virological failure (adjusted hazard ratio 3.14 [95% confidence interval 1.41–7.03, p<0.01]), whereas LLV 50–199 copies/mL was not (1.01 [0.34–4.31, p = 0.99]; median follow-up 4.5 years). LLV 200–999 copies/mL had an adjusted mortality hazard ratio of 2.29 (0.98–5.32, p = 0.05) and LLV 50–199 copies/mL of 2.19 (0.90–5.37, p = 0.09).

**Conclusions:**

In this Swedish cohort followed during ART for a median of 4.5 years, LLV 200–999 copies/mL was independently associated with virological failure. Patients with LLV had higher rates of all-cause mortality, although not statistically significant in multivariate analysis.

## Introduction

Although antiretroviral treatment (ART) improves survival and health in people living with HIV (PLHIV), mortality among PLHIV remains elevated compared to that in matched population controls [1, 2]. The reasons for this are not completely understood. In most persons receiving ART, HIV RNA cannot be detected in plasma using current routine methods with a lower limit of detection <50 copies/mL [3]. Detection of HIV RNA in plasma during ART may indicate emerging virological failure, which is variably defined as repeated HIV RNA values ≥50–1000 copies/mL [4–8], and usually with increasing viremia if treatment is not modified. A subset of patients, however, have low-level viremia (LLV), defined as repeated measurements between 20–150 copies/mL [8], 20–200 copies/ml [4], 50–200 copies/mL [5] or 50–1000 copies/mL [7].

Both the underlying cause and potential effects of LLV are unclear. LLV could be due to release of HIV particles from latently infected cells or ongoing virus replication [9]. Although LLV has been associated with an increased risk of drug resistance by some researchers, other studies suggest that this risk is low for patients with HIV RNA <50 copies/mL[10–14]. Consequently, patients with LLV may be at increased risk of virological failure [15–21]. However, other investigators have failed to identify such an association [22, 23], and the level of viremia at which the risk of virological failure becomes significant remains controversial.

Apart from the risk of drug resistance and virological failure, it has also been suggested that LLV may be associated with increased mortality [24–26]. Hitherto, studies investigating this potential association have had relatively short follow-up time (between 3 and 5 years), and this issue is hence not conclusively answered [19, 27, 28].

In this study, we aimed to investigate whether LLV is associated with a long-term risk of virological failure and all-cause mortality, respectively, in PLHIV receiving ART in Sweden.

## Methods

### Patient selection

Participants were identified from a nationwide register, InfCare HIV, that includes >99% of individuals with known HIV infection in Sweden [3]. Data was collected from the register regarding sex, date of birth, ethnicity, mode of transmission, longitudinal levels of HIV RNA, nadir CD4 cell level, antiretroviral drugs prescribed, hepatitis B and C serostatus and death. Subjects registered in the two second largest cities in Sweden (Gothenburg and Malmö) at any time point from September 1996 until October 2016 were eligible for inclusion. Patients were included at the date of the first RNA measurement after at least 12 months of triple ART. Study inclusion criteria were: age 15 years or older, prescription of ART composed of at least 3 drugs for ≥12 months and a minimum of 2 HIV RNA values ≥12 months after ART initiation. Subjects who met the criteria for virological failure, defined as 2 consecutive RNA values ≥1000 copies/mL, at this time point were excluded. All patients were followed until the date of the last clinic visit or death. Subjects who had HIV RNA results performed with an older assay with a quantification threshold of 500 copies/mL were included at the date of the first measurement with a method with a quantification limit of 50 copies/mL.

This study was conducted with ethical committee approval by the University of Gothenburg Regional Ethical Review Board (DNr: 532–11). All participants gave informed consent upon entering into the register.

### Stratification of patients

Patients were stratified into 4 groups depending on their viremia profile: 1) Permanently suppressed viremia (PSV), defined as two or more RNA values <50 copies/mL (including cases with isolated HIV RNA measurements ≥50 copies/mL followed by a consecutive suppressed value without change of treatment); 2) LLV 50–199 copies/mL (LLV-I), defined as two or more consecutive RNA values between 50–199 copies/mL, at least 1 month apart; 3) LLV 200–999 copies/mL (LLV-II), defined as two or more consecutive HIV RNA values ≥50 copies/mL, at least 1 month apart, with at least one value between 200 and 999 copies/mL; 4) High-level viremia ≥1000 copies/mL (HLV), defined as two or more consecutive HIV RNA values ≥50 copies/mL with at least one value ≥1000 copies/mL. Patients with documented interruption of treatment for one month or longer were also included in the HLV group.

### Statistical analysis

Viremia categories were correlated to two study outcomes: virological failure (defined as at least two consecutive HIV RNA measurements ≥1000 copies/mL) and all-cause death. All four categories were included in the mortality analysis, whereas only subjects with PSV, LLV-I and LLV-II were analyzed regarding the risk of virological failure. Risks of virological failure and death were analyzed using an extended Kaplan-Meier estimate, according to Snappin et al. [29]. This model accounts for the fact that patients may change group during follow-up, and updates the cohort at all event times. A Cox proportional-hazard model was fitted to assess the association of the different viremia profiles with death and virologic failure. Viremia was managed as a time-varying covariate, so that a patient could change viremia status during the follow-up time, and the cohorts were updated continuously in the analysis. Reclassification of viremia category was only done to higher viremia strata, and patients who were reclassified remained in the higher strata for the subsequent follow-up time (unless progression to a higher viremia stratum occurred). Adjustments were made for sex, age at inclusion (15–39 years, 40–59 years and ≥60 years), date of inclusion (before or after 1^st^ January 2005) and CD4 cell nadir (0–199 cells/mm^3^, 200–349 cells/mm^3^ and >350 cells/mm^3^). Since injecting drug use (IDU) is associated with greatly increased risk of death in PLHIV [30], adjustment was also made for mode of transmission (IDU or other). In order to account for potential influence on the two study outcomes of antiretroviral drug regimens that were used early during the follow-up but not are recommended today, and that may have had lower antiviral potency and/or higher rates of adverse effects, we conducted a separate sub-analysis in which only patients included 1 January 2005 or later were included. Assessment of the proportional hazard assumption was made graphically and by Schoenfeld residuals.

Baseline characteristics were compared using the nonparametric Kruskal-Wallis and Pearson’s χ^2^ tests for continuous and categorical variables, respectively. Monte Carlo simulation was used for variables with an expected cell count less than 5. Baseline characteristics comparisons were made using IBM SPSS Statistics for Windows, version 24 (IBM Corp., Armonk, N.Y., USA) and survival analyses were performed using R Statistical Software [31] with the survival [32], ggplot2 [33] and survminer packages [34].

## Results

### Patient characteristics

**Fig 1:**
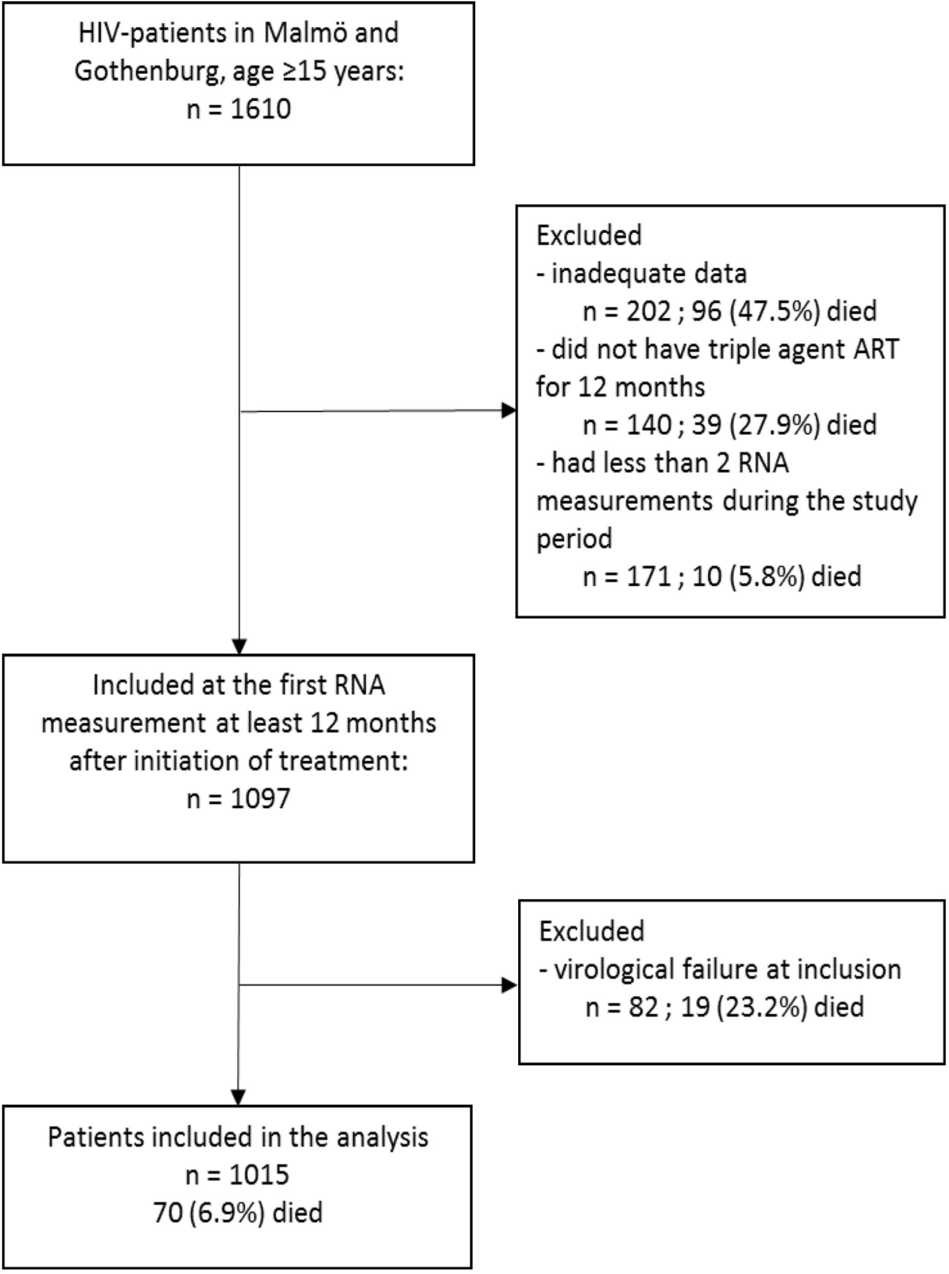
Flowchart of patient inclusion and exclusion. Abbreviations: ART–Antiretroviral treatment.

Of 1610 patients eligible for this study, 595 were excluded due to incomplete data, no registered ART or presence of virological failure at 12 months after starting ART (Fig 1). The overall mortality among excluded subjects was higher than in the 1015 patients included in the study (28% vs. 6.9%).

Baseline characteristics of included patients stratified by viremia are shown in Table 1. The median number of HIV RNA measurements per year was 2.8. Twenty-eight subjects had one or more HIV RNA results with a quantification threshold of >50 copies/mL, and were thus included at the first measurement with a more sensitive assay. Heterosexual contact was the most common route of transmission, followed by male-to-male sex. IDU was reported as route of transmission in 17 cases (1.7%).

**Table 1:** Characteristics of study subjects stratified by viremia category. Patients are categorized according to their highest viremia category during the study.

	Viremia category				
	PSV	LLV-I	LLV-II	HLV	
	n = 716 (70.5%)	n = 46 (4.5%)	n = 52 (5.1%)	n = 201 (20%)	p value*
Gender [n (%)]					0.011
Male	468 (65%)	39 (85%)	36 (69%)	148 (74%)	
Female	248 (35%)	7 (15%)	16 (31%)	53 (23%)	
Age at inclusion [median years (IQR)]	39 (33–46)	43 (35–50)	39 (34–49)	39 (33–47)	0.28
Ethnicity [n (%)]					0.0027
Caucasian	307 (43%)	25 (54%)	29 (56%)	96 (48%)	
African	172 (24%)	12 (26%)	12 (23%)	68 (34%)	
Asian	81 (11%)	2 (4.3%)	3 (3.8%)	9 (4.5%)	
Other/unknown	156 (22%)	7 (15%)	8 (15%)	28 (14%)	
Route of HIV acquisition [n (%)]					0.78
Heterosexual intercourse	370 (52%)	22 (48%)	27 (52%)	104 (52%)	
Male-to-male intercourse	264 (37%)	20 (44%)	20 (39%)	73 (36%)	
IDU	11 (1.5%)	0	1 (1.9%)	5 (2.5%)	
Blood products	20 (2.8%)	3 (6.5%)	2 (3.8%)	10 (5.0%)	
Mother-child	11 (1.5%)	0	1 (1.9%)	1 (0.5%)	
Other/unknown	40 (5.6%)	1 (2.2%)	1 (1.9%)	8 (4.0%)	
CD4 nadir [n (%)]					<0.001
0–99 cells/mm^3^	139 (19%)	13 (28%)	15 (29%)	61 (30%)	
100–199 cells/mm^3^	178 (25%)	15 (33%)	15 (29%)	66 (33%)	
200–349 cells/mm^3^	270 (38%)	16 (35%)	17 (33%)	69 (34%)	
≥350 cells/mm^3^	129 (18%)	2 (4.3%)	5 (9.6%)	5 (2.5%)	
HIV RNA at study inclusion [n (%)]					<0.001
0–199 cpm	705 (99%)	46 (100%)	39 (75%)	159 (79%)	
200–999 cpm	8 (1.1%)	0	13 (25%)	29 (14%)	
≥1000 cpm	3 (0.4%)	0	0	13 (6.5%)	
Interval between HIV diagnosis and ART initiation [median days (IQR)]	245 (24–1451)	419 (25–1524)	923 (50–2474)	725 (86–2270)	0.002
Interval between ART initiation and inclusion [median days (IQR)]	454 (410–547)	453 (402–966)	482 (390–1077)	805 (460–1618)	<0.001
Time of inclusion [n (%)]					<0.001
<1 January 2005	127 (18%)	21 (46%)	29 (56%)	150 (75%)	
≥1 January 2005	589 (82%)	24 (52%)	21 (40%)	31 (15%)	
Total time of follow-up [median days (IQR)]	1903 (911–3115)	2974 (976–4809)	2970 (1561–4927)	5089 (2830–6190)	<0.001
Number of HIV RNA measurements/year [median (IQR)]	2.71 (2.34–3.31)	3.02 (2.57–3.39)	3.49 (2.82–4.36)	3.07 (2.65–3.07)	<0.001
Hepatitis C [n (%)]					0.36
Seronegative	572 (80%)	38 (83%)	39 (75%)	150 (75%)	
Seropositive	49 (6.7%)	5 (11%)	4 (7.7%)	20 (10%)	
Unknown	95 (13%)	3 (6.5%)	9 (17%)	31 (15%)	

Abbreviations: PSV–permanently suppressed viremia; LLV-I–low-level viremia 50–199 copies/mL; LLV-II–low-level viremia 200–999 copies/mL; HLV–high-level viremia; IQR–interquartile range; IDU–injecting drug use; ART–antiretroviral treatment; cpm–copies/mL

*p values are the results of Pearson’s χ2 tests for categorical variables and Kruskal-Wallis tests for categorical variables. Monte Carlo simulation was used for variables with an expected cell count less than 5. Significance is expressed in relation to a χ distribution for the Pearson’s χ2 tests.

#### Viremia profiles at inclusion and during follow-up

**Fig 2:**
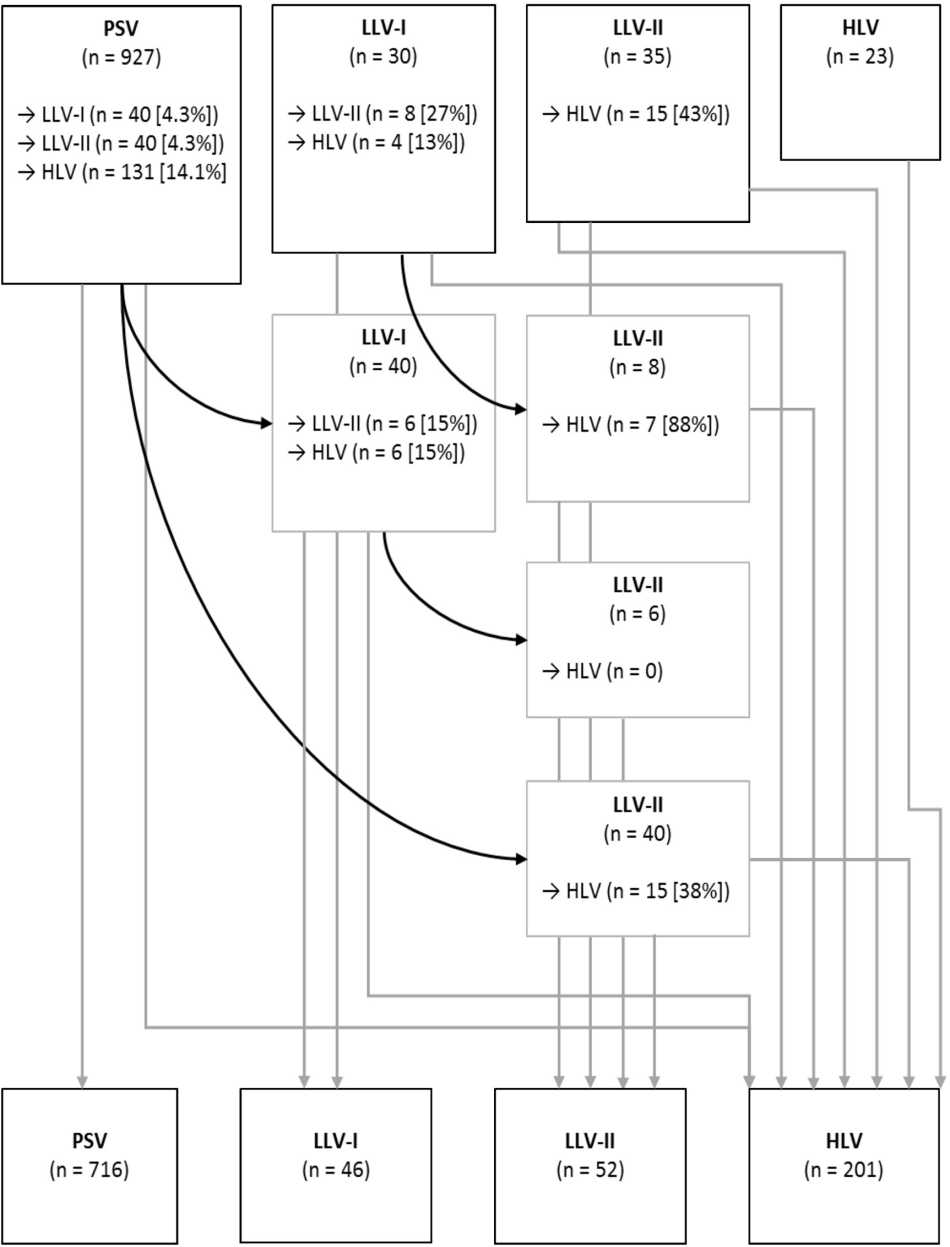
Flowchart illustrating patients changing viremia categories during follow-up. **The top of the figure represents viremia categories at inclusion and the lower part shows how patients changed categories during the follow-up time. Reclassification was only made to higher viremia strata, and the reclassified subjects remained in that strata for the remaining follow-up period (unless progression to a higher viremia stratum occurred).** Abbreviations: PSV–permanently suppressed viremia; LLV-I–low-level viremia 50–199 copies/mL; LLV-II–low-level viremia 200–999 copies/mL; HLV–high-level viremia.

Of the 1015 patients included, 927 (91%) had HIV RNA levels <50 copies/mL at inclusion, and of these 716 (77%) had persistent viral suppression. Viremia of some level was recorded during follow-up in 299 subjects (30%): 46 (4.5%) had at least one episode of LLV-I without any subsequent episode of higher level viremia, 52 (5.1%) had LLV-II without any subsequent episode ≥1000 copies/mL and 201 (20%) had HLV. In this last group, 74 had HLV despite having no documented periods without ART whereas treatment interruptions were recorded in the remaining 127 (among whom 124 later resumed ART). Progression of viremia strata during follow-up is shown in Fig 2. Proportions of subjects who progressed to a higher viremia category were greater for higher viremia levels (42% for LLV-II and 34% for LLV-I versus 23% for PSV).

Table 1 shows the distribution of patient characteristics in the different viremia categories. LLV-I was more common in men than women. Higher CD4 nadir was associated with probability of having permanently suppressed viral levels (p<0.001). Furthermore, detectable HIV RNA at inclusion was a significant predictor of future categorization into one of the higher viremia categories (p<0.001). Subjects with PSV had a higher probability of inclusion after 1 of January 2005, and had a shorter overall follow-up time (median years 5.2 [IQR: 2.5–8.5] versus 11.6 years [6.5–16.6]) compared to other viremia categories (p<0.001).

### Associations between viremia category and virological failure

**Fig 3:**
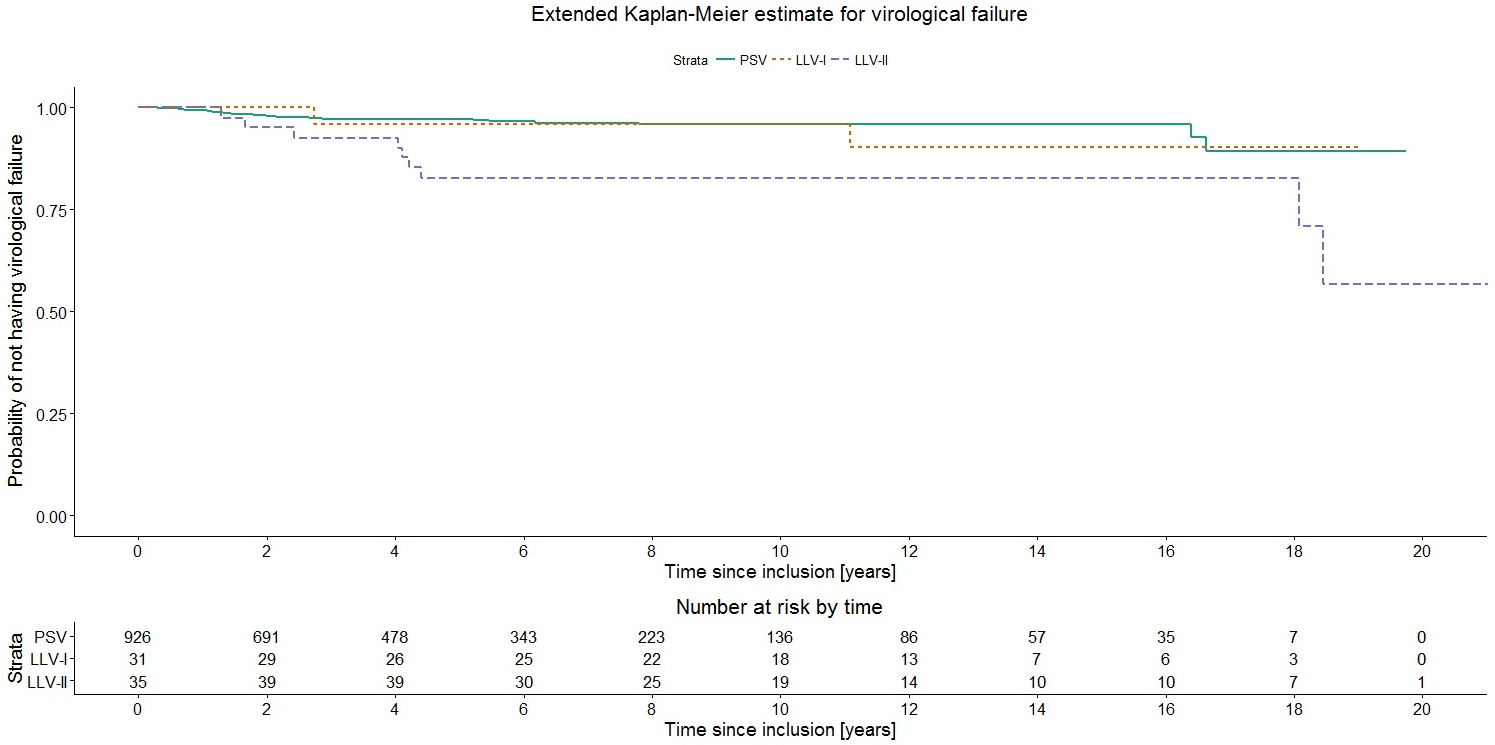
Extended Kaplan-Meier estimate for virological failure, stratified by viremia category (n = 992). Abbreviations: PSV–permanently suppressed viremia; LLV-I–low-level viremia 50–199 copies/mL; LLV-II–low-level viremia 200–999 copies/mL; HLV–high-level viremia.

During 5717 person-years of follow-up (median 4.5 years), 39 patients developed virological failure. Among these, 28 had permanently suppressed viremia prior to detection of virological failure, 2 had LLV-I and 9 had LLV-II. Based on the extended Kaplan-Meier estimate, imaginary cohorts of patients that do not change groups had a 5-year likelihood of remaining without virological failure of 0.97 (95% confidence interval [CI] 0.96–0.98) for PSV, 0.96 (95% CI 0.88–1.00) for LLV-I and 0.83 (95% CI 0.72–0.95) for LLV-II (Fig 3). The global Score test, which is comparable to the logrank test resulted in a p-value <0.01. The Cox model showed that LLV-II was associated with a significantly elevated risk of virological failure, adjusted hazard ratio (HR) 3.14 (95% CI 1.41–7.03, p<0.01), whereas LLV-I was not, adjusted HR 1.01 (95% CI 0.23–4.31, p = 0.99) (Table 2). Apart from viremia category, time of inclusion was associated with risk of virological failure, with reduced risk for patients included 2005 or later (adjusted HR 0.30 [95% CI 0.15–0.62, p<0.01]). Furthermore, the sub-analysis of 682 patients included 1 January 2005 or later showed no significant differences regarding the risk of virological failure between viremia categories (median follow-up 4.7 years; S1 Table).

**Table 2:** Crude and adjusted hazard ratios for virologic failure and all-cause death stratified by viremia category. Adjusted for sex, age at inclusion, mode of transmission (IDU vs others), time of inclusion and CD4 nadir. Results are expressed as [HR (95% CI)].

Hazard ratios for virological failure stratified by viremia category (n = 992)				
	Crude HR	p value	Adjusted HR	p value
Viremia category				
PSV	1		1	
LLV-I	1.28 (0.30–5.43)	0.74	1.01 (0.23–4.31)	0.99
LLV-II	3.98 (1.77–8.94)	<0.001	3.14 (1.17–7.03)	<0.01
Hazard ratios for all-cause death stratified by viremia category (n = 1015)				
	Crude HR	p value	Adjusted HR	p value
Viremia category				
PSV	1		1	
LLV-I	2.48 (1.02–6.00)	<0.05	2.19 (0.90–5.37)	0.09
LLV-II	2.33 (1.01–5.34)	<0.05	2.29 (0.98–5.32)	0.05
HLV	1.58 (0.90–2.78)	0.11	1.55 (0.66–3.63)	0.32

Abbreviations: HR–hazard ratio; CI–confidence interval; IDU–injecting drug use; PSV–permanently suppressed viremia; LLV-I–low-level viremia 50–199 copies/mL; LLV-II–low-level viremia 200–999 copies/mL; HLV–high-level viremia

### Association between viremia category and mortality

Seventy deaths occurred during 7812 person-years of follow-up (median 6.5 years). Causes of death are presented in Table 3.

**Table 3:** Cause of death in study subjects, in total and stratified by viremia category. Results are expressed as [n (%)].

		Viremia category			
	All subjects	PSV	LLV-I	LLV-II	HLV
	n = 70	n = 31	n = 6	n = 7	n = 26
AIDS^1^	7	3 (9.7%)	0	1 (14%)	3 (12%)
Cardiovascular disease^2^	18	10 (32%)	2 (33%)	2 (29%)	4 (15%)
Non-AIDS malignancy^3^	15	6 (19%)	1 (17%)	1 (14%)	7 (27%)
Non-AIDS infection^4^	4	3 (9.7%)	0	1 (14%)	0
Liver disease	1	1 (3.2%)	0	0	0
Pulmonary disease^5^	3	2 (6.5%)	0	0	1 (3.8%)
Violent or accidental death^6^	9	2 (6.5%)	2 (33%)	0	5 (19%)
Unknown	13	4 (13%)	1 (17%)	2 (29%)	6 (23%)

Specified causes of death

1) Diffuse centroblastic lymphoma, plasmoblastic lymphoma

2) Myocardial infarct, stroke, pulmonary edema

3) Prostate cancer, anal cancer, malignant melanoma, lung cancer, Hodgkin lymphoma, gastric cancer, pancreas cancer, squamous epithelial cancer, appendix cancer, penile cancer

4) Sepsis, infective endocarditis, pneumonia

5) Chronic obstructive pulmonary disease, pulmonary hypertension

6) Suicide, physical assault, trauma

Abbreviations: PSV–permanently suppressed viremia; LLV-I–low-level viremia 50–199 copies/mL; LLV-II–low-level viremia 200–999 copies/mL; HLV–high-level viremia

**Fig 4:**
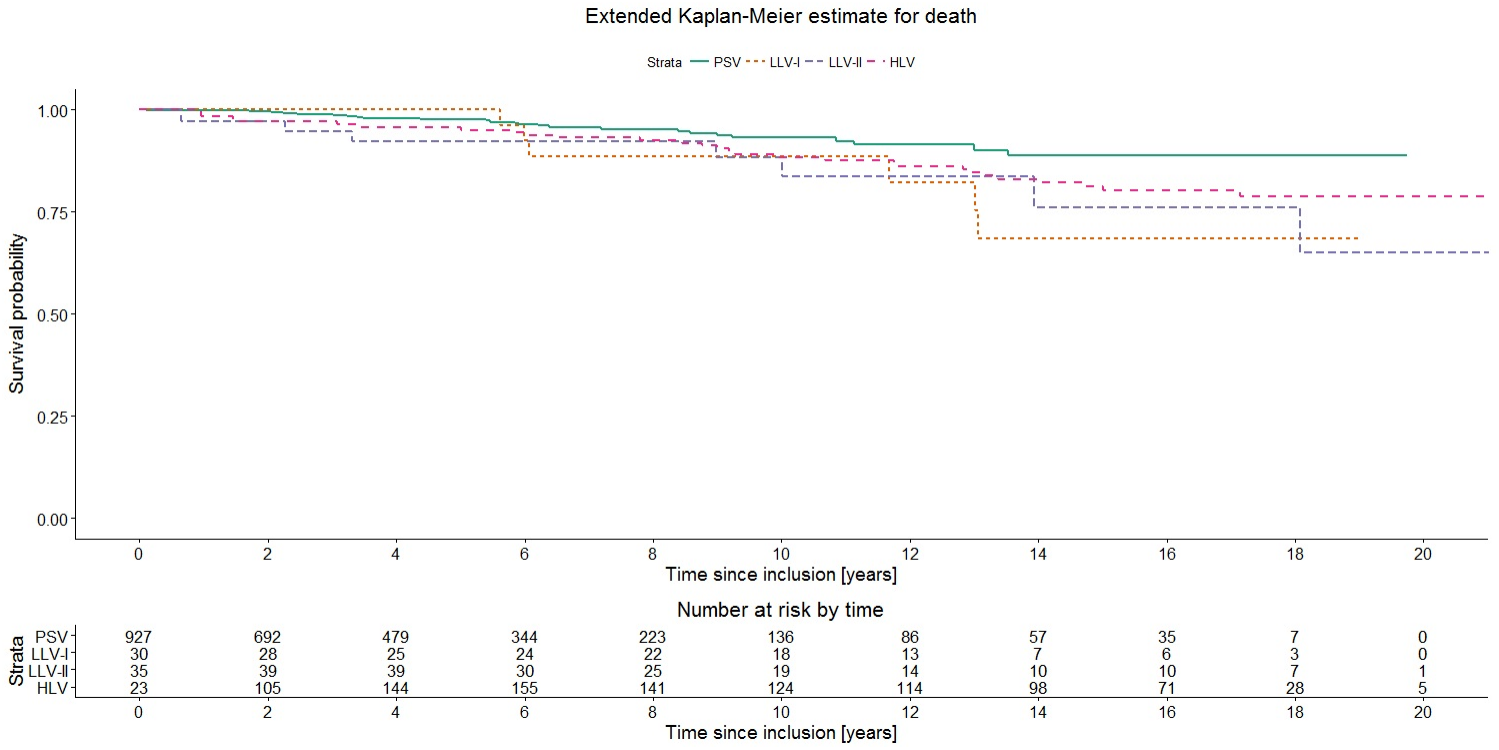
Extended Kaplan-Meier plot for death stratified by viremia category (n = 1015). Abbreviations: PSV–permanently suppressed viremia; LLV-I–low-level viremia 50–199 copies/mL; LLV-II–low-level viremia 200–999 copies/mL; HLV–high-level viremia.

Thirty-one deaths occurred in patients with PSV, 6 in patients with LLV-I, 7 in patients with LLV-II and 26 in patients with HLV. In the extended Kaplan-Meier estimate, patients with LLV had lower survival probabilities than subjects with PSV, although these differences did not reach statistical significance in the global Score test (p = 0.06) (Fig 4). In the Cox model, both LLV-I and LLV-II were associated with an elevated risk of all-cause mortality (HR 2.48 [95% CI 1.02–6.00] and 2.33 [95% CI 1.01–5.34], respectively; Table 2). After adjustment for sex, age at inclusion, mode of transmission, date of inclusion and CD4 nadir, these HRs decreased slightly (adjusted HR 2.19 [95% CI 0.90–5.37] for LLV-I, and adjusted HR 2.29 [95% CI 0.98–5.32] for LLV-II). HLV was not associated with increased mortality. Moreover, higher age at inclusion had an elevated adjusted HR for death (4.30 [95% CI 2.20–8.38] for 40–59 years and 8.86 [95% CI 3.78–20.76] for ≥60 years). Female sex was associated with decreased risk of death, but when adjusting for other risk factors, this effect was eliminated. Subjects included 1 January or later did not have a significantly reduced risk for death, compared to those included earlier (HR 0.72 [95% CI 0.41–1.25]). In a sub-analysis of 688 patients included 1 January 2005 or later no significant differences between the viremia categories regarding the risk of death were observed (median follow-up 4.7 years, S1 Table).

## Discussion

In this cohort of Swedish PLHIV, LLV in the range 200–999 copies/mL during long-term ART was associated with increased risk of virological failure. For patients with LLV in a lower range (50–199 copies/mL) this risk was not significantly elevated. We also observed higher rates of all-cause mortality in individuals with LLV; however, this difference was not statistically significant in multivariate analysis. The proportion of patients with LLV in our population was similar to that previously reported from Europe and North America [19, 35]. Although LLV during ART is a well-recognized phenomenon, its impact on treatment outcomes is not clear. Moreover, the underlying mechanisms for LLV may be heterogeneous [9], and variable definitions of LLV exist. For these reasons, the optimal clinical management of patients with LLV is not obvious.

Viral replication during ART may promote the selection of viruses carrying drug-resistance mutations, and several studies have shown that LLV <1000 copies/mL during ART is associated with drug resistance [10–12, 36]. The reported rates of drug-resistance differ between the studies, especially with regard to level of viremia. For instance, Hermanovka et al. did not find an increased risk of drug-resistance in patients with LLV <50 copies/mL [13]. In analogy, an increased risk of virological failure in patients with LLV has been reported, but methodological differences make comparisons between studies difficult, e.g. regarding the definitions of LLV and virological failure [15–17, 20, 21]. Few studies have investigated patients with LLV between 50 and 200 copies/mL [18, 19, 37]. Similar to the large Antiretroviral Therapy Cohort Collaboration-study (ART-CC) we found that the incidence of virological failure was increased in patients with LLV ≥200 copies/mL, but not for those with LLV<200 copies/mL [19]. In contrast, Laprise et al. and Vandenhende et al. reported increased risks for virological failure for patients with LLV 50–200 copies/mL (HR 2.2, 95% CI 1.60–3.09 and HR 2.30, 95% CI 1.65–3.20, respectively) [18, 37]. Three important conditions may explain these discordant results; first, whether documented viral suppression was required for inclusion; second, whether participants were required to be ART-naive or could have previous ART experience at inclusion; and finally, differences in follow-up time, which varied from 2.3 to 7.1 years. In a sub-analysis of patients included 1 January 2005 or later, the risk of virological failure was not significantly elevated among subjects with LLV in our study. Although this may be explained by a lower number of participants and few events of virological failure, this could also reflect improved potency of current antiretroviral regimens.

Since participants with LLV <200 copies/mL were reclassified to a higher viremia category in case of HIV RNA levels above this threshold during follow-up, it is possible that the risk of virological failure in lower viremia categories might have been underestimated in our study. Our data suggest that the risk of progression to a higher viremia category is higher in the LLV-I group than in patients with PSV.

Apart from virological failure we observed a trend towards increased mortality for patients with LLV. Whereas several researchers have not identified higher mortality in patients with LLV [19, 27, 28], Mugavero et al. found a strong association between the parameter viremia-copy-years (a measure of cumulative viral burden in plasma) and mortality [24]. The clinical significance of LLV might thus depend on the duration of exposure to viremia, and it is possible that long follow-up is required to identify the impact of LLV on survival. Furthermore, the level of LLV could have an impact; Quiros-Roldan et al. found that the association between viremia-copy-years and mortality was not observed in patients with low levels of viremia-copy-years (<3 log_10_ copies∙years/mL, equivalent to <200 copies/mL for 4.95 years) [38]. Although participants in our cohort were followed for a longer duration than in most of the previously mentioned studies, the association between LLV and increased mortality did not remain statistically significant after multivariate adjustment. In addition, this association was not observed in a sub-analysis of patients included 2005 or later. This is likely to be due to shorter follow-up, lower number of participants and few fatalities.

Perhaps surprisingly, viremia ≥1000 copies/mL was not associated with increased risk of death. One possible explanation is that viremia of this level prompted treatment changes resulting in better virological control and relatively low cumulative exposure to viremia. Furthermore, we included patients with documented treatment interruptions in this group, and these patients may represent a different clinical entity than subjects who have persistent viremia during ongoing ART.

Consequently, further studies are required in order to determine whether LLV leads to an increased risk of death, as well as the mechanisms involved in such an association. Chronic immune activation is recognized to be a central component of HIV pathogenesis and is linked to higher mortality [39, 40], hepatitis flares in patients coinfected with hepatitis viruses [41] and development of atherosclerosis [42]. Effective ART leads to reduced levels of plasma immune activation markers, although these levels remain higher than those in uninfected controls [43]. Several researchers have observed elevated inflammation markers in patients with LLV as compared to those with permanently suppressed replication [36, 44]. Moreover, some studies have found that ART intensification led to decreased immune activation, suggesting that persistent viral replication may be an important driver of this phenomenon in persons receiving ART [45, 46]. However, other researchers have not found such an effect of treatment intensification [47, 48], which could reflect differences both in the underlying mechanism of LLV and the link to immune activation.

Apart from the long follow-up time, a strength of our study is the statistical analysis which allowed management of LLV as a time-varying covariate. Commonly in endpoint trials, a subject’s exposure status to a risk factor may change during the follow-up; this may result in time-dependent bias if the future exposure status is analyzed as being known at inclusion. To handle this, a Cox model is typically used, with the risk factor as a time-varying covariate. Kaplan-Meier estimates usually complement Cox models in endpoint trials to facilitate the interpretation of the risk over time. Since the standard Kaplan-Meier setup does not allow time-varying covariates, we used the extended Kaplan-Meier estimate developed by Snapinn et al. [29].

Study participants were identified from a nationwide register encompassing >99% of persons diagnosed with HIV in Sweden [3]. We consider the study population to be representative for this setting, where all PLHIV receive ART through infectious disease clinics in public health care. Furthermore, the use of national identity numbers ensures minimal loss to follow-up and accurate data on mortality and emigration. Yet, incomplete data in the InfCare HIV register led to exclusion of a proportion of eligible participants. This should be considered when interpreting our results, especially since mortality in excluded cases was higher than in those included. The causes of death among our participants reflect a spectrum of diagnoses, including some unnatural fatalities. Since the cause of death could not be identified for a proportion of subjects, we chose all-cause mortality as an outcome event, instead of restricting the analysis to cases with reported natural causes of death. Our study design and the size of the cohort did not allow for analysis of the potential influence of specific antiretroviral agents on the risk of death and virological failure.

Viral load measurements were obtained from participants in clinical practice, and the sampling intervals did not allow for detailed characterizations of viremia profiles. This may have led to misclassification in some cases, especially in the group of patients with persistent viral replication, which in our study includes patients with an isolated detectable HIV RNA value. Although we interpreted these events as transient viremic episodes (blips), it is possible that some of these patients had more prolonged viremia. However, in all of these cases suppression of HIV RNA was noted in the subsequent sample without change of ART regimen. Some degree of misclassification might also have occurred due to the use of different assays for viral load determination during the study period, since intraassay correlation has been reported low for viral loads <1000 copies/mL [49].

We did not have access to data on several factors that may affect the risk of mortality. One important example is smoking, which has been found to have a great impact on mortality among PLHIV in high-income countries [50]. A nation-wide survey on HIV patients in Denmark observed other non-HIV-associated risk factors for death, such as comorbidity score, alcohol and drug use [51]. In our multivariate analysis, we adjust for the mode of transmission, which may be an indirect marker of IDU.

In conclusion, we found LLV to be associated with an increased risk of virological failure in Swedish patients receiving ART. In addition, we observed higher rates of all-cause mortality in subjects with LLV, although this association was not statistically significant in multivariate analysis. Our findings on the association between LLV 200–999 copies/mL and virological failure support current recommendations to modify therapy in such cases [4, 6]. Further research is required to investigate whether LLV is associated with increased mortality.

## Supporting information

S1 Table Sub-analysis with subjects included 1 January 2005 or later. **Adjusted for sex, age at inclusion, mode of transmission (IDU vs others) and CD4 nadir. Results are expressed as [HR (95% CI)].** Abbreviations: HR–hazard ratio; CI–confidence interval; IDU–injecting drug use; PSV–permanently suppressed viremia; LLV-I–low-level viremia 50–199 copies/mL; LLV-II–low-level viremia 200–999 copies/mL; HLV–high-level viremia; N/A–not applicable
(DOCX)
