# Supplementary material for: Virological failure and all-cause mortality in HIV-positive adults with low-level viremia during antiretroviral treatment
Source: PLoS One. 2017 Jul 6;12(7):e0180761. doi: 10.1371/journal.pone.0180761 (PMC5500364; doi:10.1371/journal.pone.0180761)
Supplement: S1 Table — Adjusted for sex, age at inclusion, mode of transmission (IDU vs others) and CD4 nadir. Results are expressed as [HR (95% CI)]. Abbreviations: HR–hazard ratio; CI–confidence interval; IDU–injecting drug use; PSV–permanently suppressed viremia; LLV-I–low-level viremia 50–199 copies/mL; LLV-II–low-level viremia 200–999 copies/mL; HLV–high-level viremia; N/A–not applicable (DOCX) [file pone.0180761.s001.docx]

**S1 Table. Sub-analysis with subjects included 1 January 2005 or later. Adjusted for sex, age at inclusion, mode of transmission (IDU vs others) and CD4 nadir. Results are expressed as [HR (95% CI)].**

| Hazard ratios for virological failure stratified by viremia category (n=682) | | | | | |
| --- | --- | --- | --- | --- | --- |
|  | | Crude HR | p value | Adjusted HR | p value |
| Viremia category | |  |  |  |  |
|  | PSV | 1 |  | 1 |  |
|  | LLV-I | N/A | 1.00 | N/A | 1.00 |
|  | LLV-II | 3.23 (0.41–25.7) | 0.27 | 3.09 (0.36–26.5) | 0.30 |
| Hazard ratios for all-cause death stratified by viremia category (n=688) | | | | | |
|  | | Crude HR | p value | Adjusted HR | p value |
| Viremia category | |  |  |  |  |
|  | PSV | 1 |  | 1 |  |
|  | LLV-I | 2.21 (0.29–17.0) | 0.45 | 2.07 (0.26–16.5) | 0.49 |
|  | LLV-II | N/A | 1.00 | N/A | 1.00 |
|  | HLV | 2.28 (0.62–8.32) | 0.21 | N/A | 1.00 |

Abbreviations: HR – hazard ratio; CI – confidence interval; IDU – injecting drug use; PSV – permanently suppressed viremia; LLV-I – low-level viremia 50–199 copies/mL; LLV-II – low-level viremia 200–999 copies/mL; HLV – high-level viremia; N/A – not applicable
